# Medico-Legal

**Published:** 1910-01

**Authors:** John A. Rafter


### PROGRESS IN MEDICAL SCIENCE.

### Medico-Legal

CONDUCTED by JOHN A. RAFTER, M. D.

### LEGAL RIGHTS OF DOCTORS.

THE physician's professional and legal obligations are parallel in most cases. He need not attend every patient who applies for treatment, but having assumed charge of a case he is required to continue his treatment as long as is necessary, and to exercise
ent is a charity patient or no, he must bring the requisite skill
ent is a charity patient or not, he must bring the requisite skill
and knowledge to a case. He must exercise care in visiting patients ill from a contagious disease. If he uses his best judgment he is doing all that the law requires. In case of an operation on a wife who has consented to it, the consent of the husband is not required. An autopsy should not be performed without the consent of the next of kin. The question of fees is decided by the fees generally charged in the locality where the physician is practising. In order to obtain his fees he must show that he is a legally qualified physician, and must prove his license to practise.

---

DO NOT GUARANTEE A CURE!

In order to avoid suits for malpractice, the loss of time and the annoyance entailed by such suits, no matter what the termination may be, it is most important that physicians should never guarantee or contract to cure even of the simplest kind. Actions for damages are now so often brought against even the most distinguished of the profession, it is well to be guarded in stating the probable outcome of any case of injury or for that matter any case of whatsoever kind. The law will not accept any excuse as to why the practitioner did not fulfil his contract.

---

ALWAYS GET CONSENT.

When an injury has occurred and an operation is deemed necessary for its correction, it should always be the rule to obtain the consent of the patient, to do whatever is necessary after the anesthetic is administered. In case the patient is not in a condition to understand, or is a minor, the consent of some responsible member of the family should be obtained. If one is called to a case where amputation of a limb is necessary—and should the patient refuse to be operated upon, his wishes should be respected—a suit for malpractice would likely follow if any other course was adopted.

---

ANTEMORTEM STATEMENTS.

The simple statement of an injured person who thinks he will recover when the statement is made but who afterward dies as the result of the injury, is of very little value in court. When a physician is called to see an individual dying as the result of accident, assault or of an attempt at self-destruction and who is capable of making an antemortem statement, he should be asked to do so. But before the declaration is taken, the patient should be informed that he has no chance to recover and that death is certain. His statements then whatever they are, should be written in the exact language used and if possible in the presence of witnesses. Statements of this kind are now accepted by the courts without being sworn to.

---

PERTAINING TO PARTNERSHIPS.

In partnerships among physicians no legal responsibility is assumed by one for the other, so far as the medical or surgical practice is concerned. In a suit for malpractice against a practitioner where a judgment is secured, a partner who had nothing to do with the case would not be liable for errors in the professional work of his associate. In case of any commercial transactions where business outside of ordinary professional work is conducted each would be responsible to the same extent as in other business concerns.

